# Molecular and Structural Characterization of the Tegumental 20.6-kDa Protein in *Clonorchis sinensis* as a Potential Druggable Target

**DOI:** 10.3390/ijms18030557

**Published:** 2017-03-04

**Authors:** Yu-Jung Kim, Won Gi Yoo, Myoung-Ro Lee, Jung-Mi Kang, Byoung-Kuk Na, Shin-Hyeong Cho, Mi-Yeoun Park, Jung-Won Ju

**Affiliations:** 1 Division of Malaria and Parasitic Diseases, Centre for Immunology and Pathology, Korea National Research Institute of Health, Chungbuk 28159, Korea; hoiyui25@gmail.com (Y.-J.K.); wongi.yoo@gmail.com (W.G.Y.); blackcg96@gmail.com (M.-R.L.); jo4u@cdc.go.kr (S.-H.C.); miyeoun@korea.kr (M.-Y.P.); 2 Department of Parasitology and Tropical Medicine, and Institute of Health Sciences, Gyeongsang National University School of Medicine, Jinju 52727, Korea; gjm9951001@hanmail.net (J.-M.K.); bkna@gnu.ac.kr (B.-K.N.)

**Keywords:** *Clonorchis sinensis*, tegument, homology modelling, I-TASSER, structure, localization, virtual screening, docking, compound

## Abstract

The tegument, representing the membrane-bound outer surface of platyhelminth parasites, plays an important role for the regulation of the host immune response and parasite survival. A comprehensive understanding of tegumental proteins can provide drug candidates for use against helminth-associated diseases, such as clonorchiasis caused by the liver fluke *Clonorchis sinensis*. However, little is known regarding the physicochemical properties of *C. sinensis* teguments. In this study, a novel 20.6-kDa tegumental protein of the *C. sinensis* adult worm (CsTegu20.6) was identified and characterized by molecular and in silico methods. The complete coding sequence of 525 bp was derived from cDNA clones and encodes a protein of 175 amino acids. Homology search using BLASTX showed CsTegu20.6 identity ranging from 29% to 39% with previously-known tegumental proteins in *C. sinensis*. Domain analysis indicated the presence of a calcium-binding EF-hand domain containing a basic helix-loop-helix structure and a dynein light chain domain exhibiting a ferredoxin fold. We used a modified method to obtain the accurate tertiary structure of the CsTegu20.6 protein because of the unavailability of appropriate templates. The CsTegu20.6 protein sequence was split into two domains based on the disordered region, and then, the structure of each domain was modeled using I-TASSER. A final full-length structure was obtained by combining two structures and refining the whole structure. A refined CsTegu20.6 structure was used to identify a potential CsTegu20.6 inhibitor based on protein structure-compound interaction analysis. The recombinant proteins were expressed in *Escherichia coli* and purified by nickel-nitrilotriacetic acid affinity chromatography. In *C. sinensis*, CsTegu20.6 mRNAs were abundant in adult and metacercariae, but not in the egg. Immunohistochemistry revealed that CsTegu20.6 localized to the surface of the tegument in the adult fluke. Collectively, our results contribute to a better understanding of the structural and functional characteristics of CsTegu20.6 and homologs of flukes. One compound is proposed as a putative inhibitor of CsTegu20.6 to facilitate further studies for anthelmintics.

## 1. Introduction

*Clonorchis sinensis* is an endemic trematode parasite that causes human clonorchiasis. It is estimated that approximately 35 million people are infected with this fluke in East Asia, including Korea, China and Vietnam [1,2,3]. Individuals generally become infected with *C. sinensis* by the consumption of raw or undercooked freshwater fish containing metacercariae [4]. The *C. sinensis* adult worms inhabit the bile ducts and cause a series of pathological changes, such as epithelial hyperplasia, periductal fibrosis, obstructive jaundice, dyspepsia and liver cirrhosis, in the infected hosts [3]. Chronic clonorchiasis has been classified as a definite biological carcinogen that causes cholangiocarcinoma in humans by the World Health Organization [5].

The recommended treatment of clonorchiasis is the chemotherapeutic application of praziquantel, utilized for both the treatment and control of clonorchiasis [6,7]. Praziquantel is also required for the treatment of other intestinal trematode infections. However, it has been reported that praziquantel exhibits low efficacy for the treatment of clonorchiasis in northern Vietnam [8]. As dependency on a specific drug would be ineffective for any human trematode infections, novel anti-clonorchiasis compounds should therefore be developed to ensure continued or enhanced management of trematode-associated diseases.

Helminth tegumental proteins have raised interest as both a diagnostic and potentially druggable therapeutic target [9,10], as they are essential for establishing the host and parasite relationship. The tegument, which covers the entire surface of the worm, comprises a unique outer surface syncytium structure that is of crucial importance for nutrient uptake, excretion, osmoregulation, sensory and signal transduction, host response modulation and parasite survival [11,12]. To date, five genes encoding *C. sinensis* tegumental proteins have been identified and characterized; additionally, their potential as diagnostic antigens for clonorchiasis has been evaluated [13,14,15,16,17]. However, their druggability has yet to be assessed.

In the present study, we identified and characterized the tegumental protein of 20.6 kDa in *C. sinensis* (CsTegu20.6). Structure and functional analyses were carried out using combined three-dimensional (3D) modeling methods. Computer-aided drug discovery (CADD), such as virtual inhibitor screening and drug-likeness prediction, was used to identify a potent inhibitor of compound interactions with CsTegu20.6.

## 2. Results and Discussion

### 2.1. Physico-Chemical and Functional Characterization

A cDNA clone representing the complete sequence CsTegu20.6 was isolated using the *C. sinensis* adult cDNA library [18]. Sequence analysis of CsTegu20.6 indicated an open reading frame (ORF) of 528 nucleotides, and the deduced amino acid (aa) sequence revealed a protein of 175 residues with a calculated molecular mass of 20.57 kDa and a theoretical isoelectric point (pI) of 6.15. This protein was predicted to be localized in the cytoplasm and did not contain any signal peptides or transmembrane domains. A functional domain search indicated the presence of an EF-hand domain (PS00018) at aa46–58 and a dynein light chain domain (DLC) (PF01221) at the C-terminus (aa97–173) (Figure S1). This is in broad accordance with earlier work in which tegumental proteins were analyzed using bioinformatics tools and shown to be composed of one or two EF-hand domains and a DLC domain at the N- and C-termini, respectively [13,17].

### 2.2. Sequence-Based Similarities

**Figure 1:**
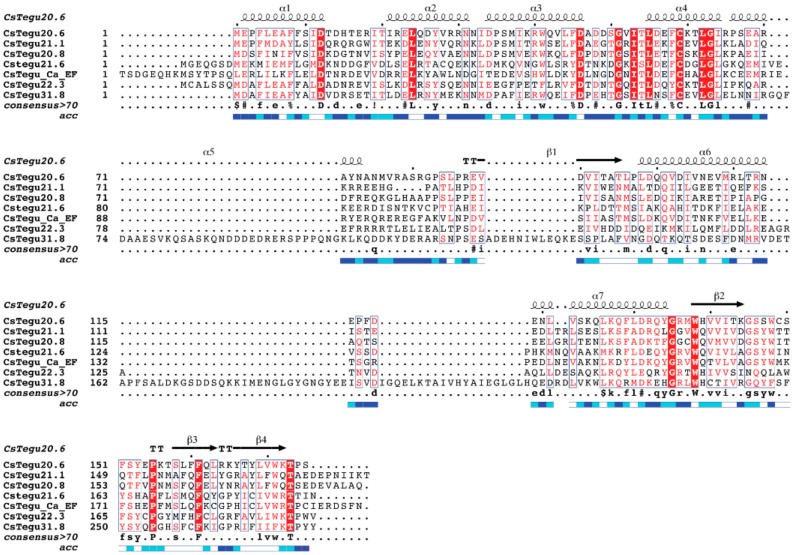
Comparison of amino acid sequence of the 20.6-kDa tegumental protein of *C. sinensis* (CsTegu20.6) with other *C. sinensis* tegumental proteins. Multiple sequences alignment was visualized by ESPript [21] after alignment of the protein sequences using MAFFT [22]. Secondary structure features of CsTegu20.6 are given above the alignments. α-helices and β-strands are represented as helices and arrows, respectively, and β-turns are marked with TT. Conserved areas are shown shaded. Conserved sequences are indicated by a box if more than 70% of the residues are similar. The similar sequences are indicated by colored background considering physico-chemical properties. “acc” indicates the relative accessibility of each residue. The blue square scale is set as follows: “accessible” (**blue**, 0.4 < A ≤ 1.0), “intermediate” (**cyan**, 0.1 ≤ A ≤ 0.4) and “buried” (**white**, A < 0.1). Accession numbers of the sequences presented are as follows: CsTegu21.1 (ADZ13689.1), CsTegu20.8 (ABC47326.1), CsTegu21.6 (JF911532), CsTegu_Ca (ABZ82044), CsTegu22.3 (ABK60085.1) and CsTegu31.8 (ABK60086.1).

When CsTegu20.6 was compared with six other tegumental proteins of *C. sinensis*, multiple sequences alignment of the amino acids revealed that CsTegu20.6 shares 39% identity with CsTegu21.1 (NCBI Accession No. ADZ13689.1) [13], 38% with CsTegu20.8 (ABC47326.1) [15], 29% with CsTegu21.6 (JF911532) [17], 38% with CsTegu_Ca_EF (ABZ82044), 32% with CsTegu22.3 (ABK60085.1) [16] and 35% with CsTegu31.8 (ABK60086.1) [14] (Figure 1). Both the consensus sequences (Asp46, Gly51, Ile53, Leu55, Cys59, Leu62 and Gly63, shaded in red) and the conserved helix-loop-helix (HLH) motif were observed in the calcium-binding EF-hand domain. The HLH structure is an important characteristic of EF-hand domains, of which residue mutation in the loop region can cause an inability of calcium-binding activity [19]. The length of the EF-hand of CsTegu20.6 was almost similar to that of CsTegu21.6, which was conspicuously shorter than those of other *C. sinensis* tegumental proteins [17]. The DLC domain was less conserved than the EF-hand domain. Although the functions of the DLC domain remain unclear, DLC appears to act as part of a large complex and to contribute to maintenance of the tegument [20].

### 2.3. Improved and Full-Length 3D Models Using a Combined Approach

We attempted to predict the 3D model of CsTegu20.6 based on homology modeling using Swiss-Model [23]. However, the first attempt failed because the proper template structures were not found in the protein data bank (PDB) template library, as experimental structures of helminth tegumental proteins were not available. The search for templates showed a very low percentage of sequence identities (<25%) and coverages (<51%). We next attempted to predict a CsTegu20.6 model based on multiple-threading alignments using I-TASSER [24]. The 3D model was constructed using 10 multiple templates and refined with energy minimization. However, the second attempt also was not successful because the Ramachandran plot for the model showed 64.8% residues in the most favored regions and 3.8% residues in the disallowed regions (Figure S2).

**Figure 2:**
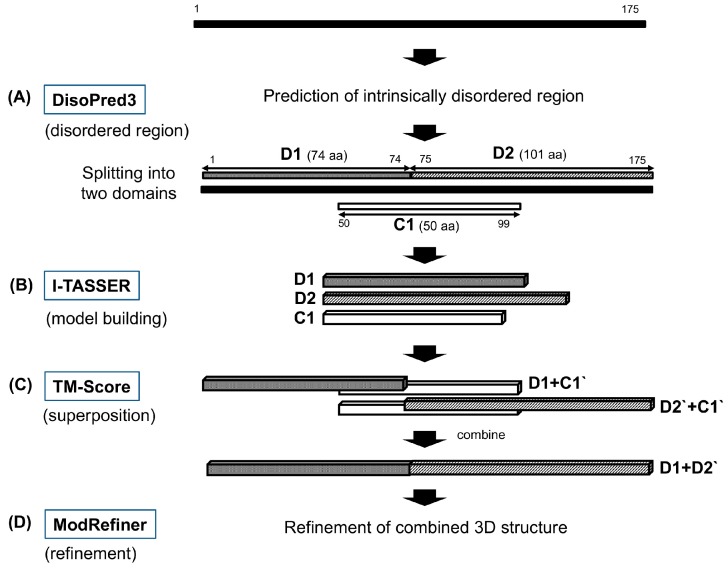
Workflow for the full-length and refined structure of CsTegu20.6.

To improve the quality of the full-length CsTegu20.6 model, we then used combined 3D modeling methods and refinement as described in Section 3.2. (Figure 2). It should be noted that the disordered region was placed between Thr61 and Asp90 (Figure S3). This region can decrease the quality of the overall model, as it requires additional simulation time, and it interferes with the structural clustering process [25]. These regions have been predicted representing tegumental protein linkers because they lack secondary structure in *Schistosoma mansoni* [26] and *Fasciola hepatica* [27]. To address this issue, we split the whole sequence into two domains, domain 1 (aa1–74) and domain 2 (aa75–175), based on the disordered region rather than removing the region. The model of each domain was constructed using I-TASSER [24] based on iterative fragment assembly simulations. Then, the structures of the unaligned regions, as for the disordered region, were constructed by ab initio modeling based on replica-exchange Monte Carlo simulations [28]. The structures of the two domains were finally combined into a full-length model using the C1 fragment aa50–99 as a bridge using TM-Scores [29]. The initial model of CsTegu20.6 was obtained by refining the combined model using ModRefiner [30] (Figure S4). This program is designed to improve the physical realism and structural quality of protein models via two-step atomic-level energy minimization. Because ModRefiner can build the coordinates of the unfolded regions, the program has been employed as a key method of the combined approach [31].

**Figure 3:**
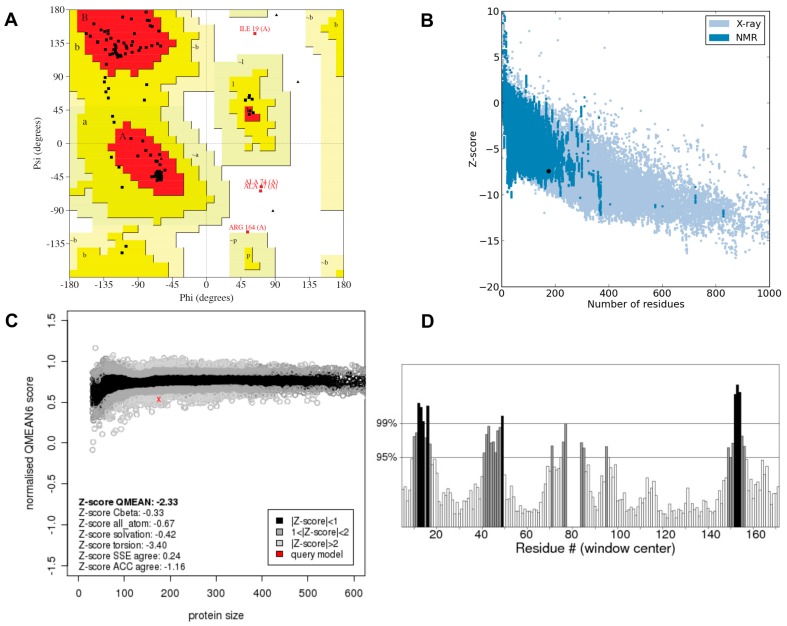
Validation of the CsTegu20.6 3D model. (**A**) The Ramachandran plot shows the residue in most favored regions (85.5%), additional allowed regions (11.9%), generously allowed regions (0.1%) and disallowed regions (2.5%). Red (A, B, L), yellow (a, b, l, p) and light yellow (~a, ~b, ~l, ~p) indicate the most favored regions, allowed regions and generously allowed regions. White shows disallowed regions. All non-glycine and non-proline residues are shown as closed black squares while glycines (non-end) are shown as closed black triangles. Disallowed residues are colored in red; (**B**) the ProSA energy profile indicates that the Z-score was −7.41; (**C**) the QMEAN Z-score is −2.33; (**D**) in the ERRAT plot, the overall quality factor is 80.75%.

The final model of CsTegu20.6 was determined after the loops and both main- and side-chains of the initial model were further refined using GalaxyLoop [32] and GalaxyRefine [33], respectively (Figure 3). The ab initio modeling method, hybrid-type energy function and an efficient search method were employed within GalaxyLoop. Then, both the backbone and side chain of the structure were refined using GalaxyRefine based on repeated perturbation and, hence, overall conformational relaxation by short molecular dynamics simulations. Through the refinement procedure of the initial model, the stereochemical quality of the final model was markedly improved over that of the initial model, especially in terms of the ERRAT values from 48.5% (Figure S3D) to 80.8% (Figure 3D). ERRAT calculates the overall quality score of non-bonded atomic interactions compared with the database of reliable experimental structures.

### 2.4. Structure Validation

The Ramachandran plot [34], ProSA [35], QMEAN [36], ERRAT [37] and ModFOLD6 [38] methods were employed to validate the final model of CsTegu20.6. The Ramachandran plot ensured the quality of the final model, indicating that 85.5% of residues were in the favorable region and 11.9% in the additionally allowed region (Figure 3A). Furthermore, there were only 2.5% residues in the disallowed region, and 0% of the residues were located in the generously allowed region. Only Ile19, Ala47, Ala74 and Arg164 were found in the disallowed region of the plot. This plot was used to assess the constructed model for its main chain conformation, illustrating the Φ-Ψ torsion angles for all residues, except for Gly and Pro. The ProSA Z-score of −7.41 confirmed the final model as an extremely good model (Figure 3B), whereas the QMEAN Z-score of −2.33 suggested that the model might be a fairly good representation of the protein (Figure 3C). ProSA analyzed the overall quality score by calculating the atomic coordinates of the model, indicating the Z-score of the experimentally solved structures deposited in PDB [39], whereas the QMEAN Z-score is a measure of the “degree of nativeness” of a given structure and provides information regarding whether the model is of sufficient quality to be comparable to high-resolution crystal structures of similar size. The ERRAT overall quality score of 80.8 showed that the structure could be considered as a good model (Figure 3D). The ModFOLD6 global model quality score of 0.49 indicated that the final model was confidently folded, and the 3D cartoon of the model was colored by the per-residue error according to the B-factor values (Figure S5).

### 2.5. Structure-Based Similarity

**Figure 4:**
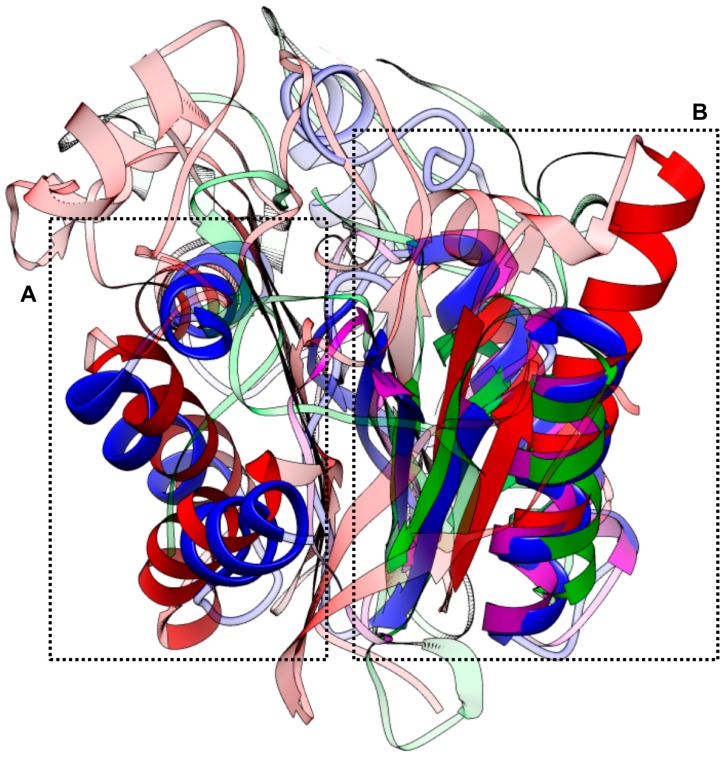
The structure-based similarity and superposition. Three homologues were superposed on to CsTegue20.6 using the deconSTRUCT [40]. The superposed output is represented by the solid color, while the remainder of the structures is semi-transparent. The CsTegu20.6 (**blue**) was aligned to the calcium-binding protein (**green**, PDB ID: 1JFJ_A) on 37.1% (65/175) (**A**) and also both dynein light chain 1 (**red**, PDB ID: 1YO3_A) and dynein light chain 2 (**purple**, PDB ID: 1RE6_A) on 44.6% (78/175) (**B**).

The results of the structure-based search for homologues using deconSTRUCT [40] showed that CsTegu20.6 has the highest homology with several DLCs, followed by ribosomal proteins and calcium-binding proteins (Table 1). We superposed the structures of two DLCs (PDB IDs: 1YO3_A and 1RE6_A) (Figure 4B) and the calcium-binding protein (PDB ID: 1JFJ_A) (Figure 4A). The superposition of structures confirmed that (1) the proper templates were not available for whole structure and partial templates were aligned on the N- and C-termini; and (2) the structural and functional aspects of each domain can be interpreted because of high degrees of conservation.

### 2.6. Overall Structural Features and Dimerization

**Figure 5:**
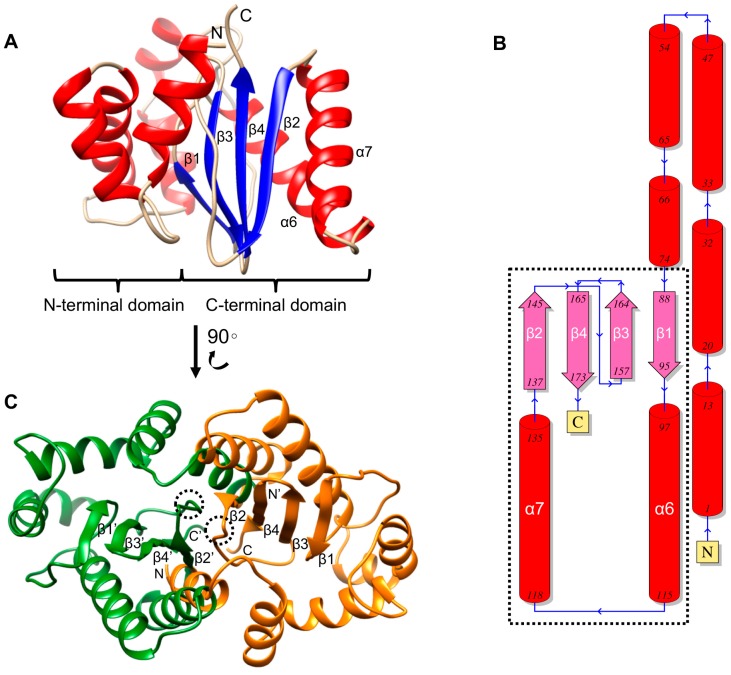
Structural characterization and homodimer. (**A**) The α-helices, β-strands and coils of the CsTegu20.6 are colored in red, blue and gold, respectively. N- and C-terminal domains are connected by a flexible linker, which is an intrinsically disordered region. The C-terminal domain constitutes a sandwich of a four-stranded antiparallel β-sheet, β1-β3-β4-β2, and two α-helices, α6 and α7; (**B**) Secondary structure topology diagram showing domain composition and connectivity. The ribbon diagram indicates α-helices and β-strands in red and magenta color, respectively. The diagrams associated with the ferredoxin fold are indicated with a dotted rectangle; (**C**) The homodimer with symmetry-related CsTegu20.6 monomers (**green** and **orange**) was constructed by GalaxyGemini [44]. The missed fifth β-strand is indicated with dotted circles [45] (see Section 2.6).

The final model of CsTegu20.6 consisted of two domains: a N-terminal domain (calcium-binding domain) and a C-terminal domain (DLC domain) adopting a classic ferredoxin fold [41], which forms a four-stranded antiparallel β-sheet flanked on one side by two α-helices (Figure 5A,B). However, unlike a typical ferredoxin fold associated with the overall symmetry of β-α-β-β-α-β, the β1-α5-α6-β2-β3-β4 fold was present in the DLC domain of CsTegu20.6. The two α-helices, α6 and α7, were packed in an antiparallel hairpin structure against the β-sheet with four antiparallel β-strands, β1-β3-β4-β2. These findings are in line with structural data obtained from a DALI search [42] that found similarities to numerous proteins with DLC1 (also known as DLC8), such as 5E0M and 3DVT, the structures of which represent the same fold [43]. DLC-containing proteins can form homodimers through hydrogen bonds, backbone-side chain electrostatic interactions and van der Waals contacts between side chains [43]. Previous studies have indicated that the homodimeric DLCs in *S. mansoni* [26] and *F. hepatica* [27] were composed of two asymmetric monomers through β-sheet interactions. The homodimer of CsTegu20.6 was constructed using GalaxyGemini [44] with only the C chain of DLC1 (PDB ID: 4DS1_C), although the fifth β-strand (indicated with a dotted circles) as a monomeric counterpart was missed in the predicted structure [45] (Figure 5C). This result suggests that CsTegu20.6 can dimerize, confirming that this feature is conserved between the DLCs.

### 2.7. EF-Hand Calcium-Binding Site

**Figure 6:**
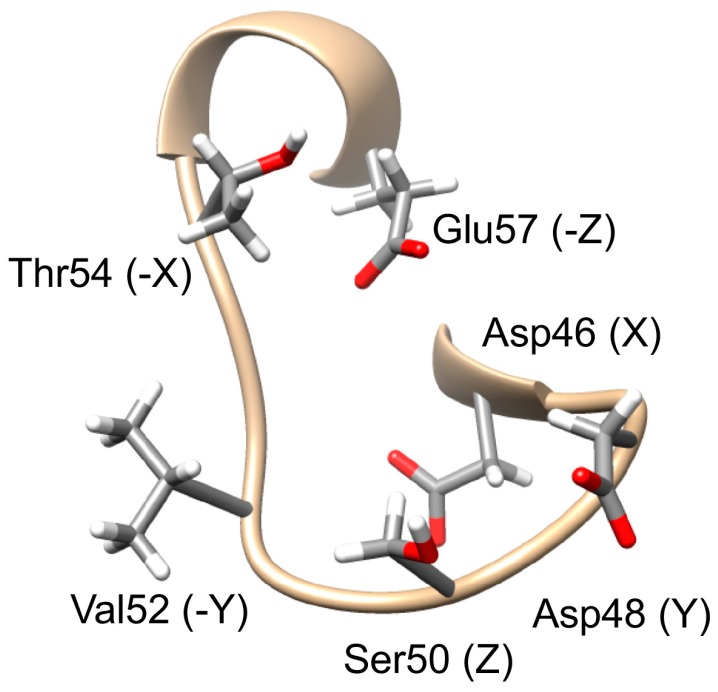
EF-hand calcium-binding site. The Ca^2+^-binding site of CsTegu20.6 was predicted from that of *Rattus norvegicus* (PDB ID: 1XVJ_A). The consensus binding residues are Asp46, Asp48, Ser50, Val52, Thr54 and Glu57, and the labels are indicated in parentheses. Side-chain oxygen and hydrogen atoms were colored in red and white, respectively.

In a typical EF-hand, six residues are necessary for the coordination of the calcium ion. These are labeled as the X, Y, Z, −Y, −X and −Z residues, and consensus residues at these positions have been identified [46]. Residues Asp46, Asp48, Ser50, Val52, Thr54 and Glu57 were predicted as constituting a potential calcium-binding site in the EF-hand of CsTegu20.6 using COACH [47] (Figure 6). The consensus is mostly observed, with a different residue, Val52, at the −Y residue, which is rarely observed at this position. The orientation of the Val52 side chain is also more distant from the calcium-binding site compared to that of the Thr52 side chain in a typical EF-hand. Therefore, it is improbable that this EF-hand contacts calcium ions.

### 2.8. Virtual Inhibitor Screening

Screening for putative inhibitors of CsTegu20.6 was performed using GalaxySite [48], which was employed to predict compounds and binding conformations for CADD. The template search was performed based on experimental structures of template proteins complexed with compounds in the local alignment mode and then was optimized using the conformational space annealing algorithm. Six putative inhibitors were identified: STU, ANP, BK3, CRK, DTQ and MRD (Table S1).

**Figure 7:**
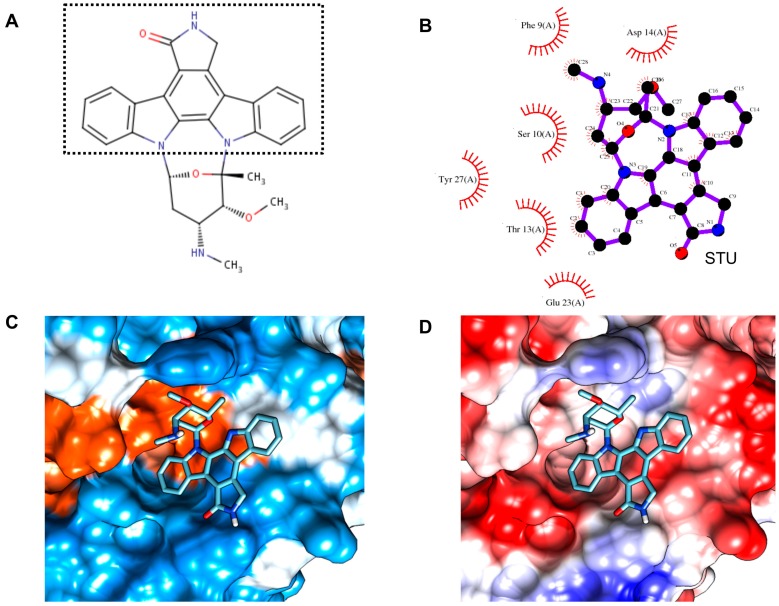
Structural and detailed view of interaction of compound with CsTegu20.6. (**A**) STU (staurosporine). The fused indole and carbazole ring is indicated with a dotted rectangle; (**B**) The binding mode of STU in the CsTegu20.6 binding pocket obtained from GalaxySite [48]. Putative inhibitor and residues that are in close contact with each other are indicated in 2D diagrams. The residues, marked with red spoked arcs, involved in hydrophobic interactions with the compound; (**C**) Hydrophobic surface. The binding pocket is colored from blue for the hydrophilic region to orange for the hydrophobic region; (**D**) Electronic surface. The binding pocket is indicated from blue for the negatively-charged region to red for the positively-charged region.

After the two compounds STU and ANP were further screened by predicting the absorption, distribution, metabolism, excretion and toxicity (ADMET) properties of all compounds using admetSAR [49], only STU (staurosporine) was found to be acceptable according to Lipinski’s rule of five [50] (Table S1). The absorption factors for STU resulted in high probabilities (97.3%) for human intestinal absorption, but moderate probabilities for blood brain barrier and CaCo-2 permeability. Under the metabolism profile, STU was found to be a substrate only for cytochrome P450 (CYP450) 3A4, a non-inhibitor for all different forms of CYP450, thus having high CYP inhibitory promiscuity. Under the toxicity profile, STU demonstrated the absence of mutagenic toxicity (a.k.a. AMES toxicity) and carcinogenic effects and also was found to be not toxic for fish and honey bee. Collectively, these data suggest that STU represents a safe and orally-absorbed drug candidate (Figure 7A), whereas the other compounds were ruled out for reasons, such as toxicity for fish (BK3 and CRK), AMES toxicity (DTQ), carcinogenicity (MRD) and violations of Lipinski’s rule (hydrogen bond acceptors, hydrogen bond donors and molecular weight for ANP) (Table S1).

To further investigate the spatial characteristics of the compound binding properties, the protein-compound interaction was analyzed. The associated binding pocket appeared shallow and open in shape. Hydrophobic (Phe9, Thr13 and Tyr27) and electronegative (Asp14 and Glu23) regions were found in the center and wall of the pocket, respectively (Figure 7C,D). One-half of the fused indole and carbazole ring of STU was inserted in the pocket, whereas the rest of the compound was located in the shallow groove. Although no typical hydrogen bonds were identified, STU can tightly bind to CsTegu20.6, resulting in a docking energy of −7.23 kcal/mol (Figure 7B and Table S1). STU, a well-known protein kinase inhibitor, has previously been proposed as an effective drug for protozoan parasites [51,52]. Its analogues also showed strong inhibitory effects against a number of infectious agents, including antibacterial and immunosuppressive activities [53]. These studies, therefore, provide speculative justification that STU might exhibit the potential to become a leading putative anti-clonorchiasis compound.

### 2.9. Production of Recombinant CsTegu20.6

**Figure 8:**
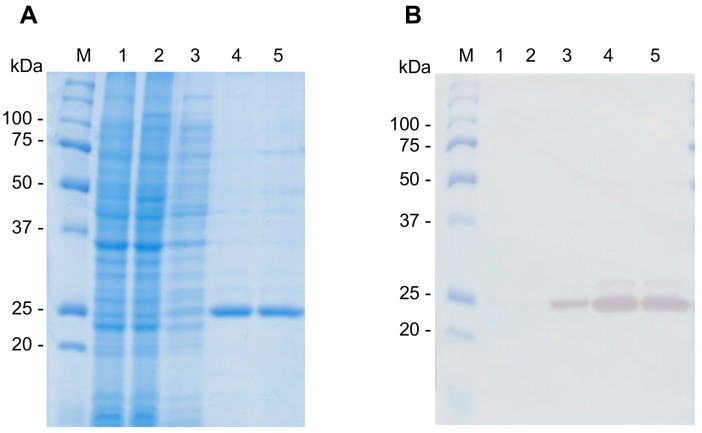
Expression and purification of rCsTegu20.6. The rCsTegu20.6 was detected in *E. coli* BL21(DE3) pLysS through SDS-PAGE staining by Coomassie brilliant blue (**A**) and Western blot with anti-polyhistidine antibody (**B**). Molecular weight marker (M), lysate of *E. coli* with pRSETb-CsTegu20.6 before induction (Lane 1) and after induction (Lane 2), flow through (Lane 3), purified recombinant proteins (Lanes 4 and 5).

Recombinant (r)CsTegu20.6 was purified under native conditions using a histidine tag at the N-terminus. The observed molecular weight was consistent with the predicted molecular mass (Figure 8A). The purified protein was verified using a monoclonal anti-His antibody (Figure 8B). The liquid chromatography-mass spectrometry performed according to our previously reported method [54] identified six specific peptide fragment sequences: _31_NNIDPSMIKRWQVLFDADDSGVITLDEFCK_60_, _41_WQVLFDADDSGVITLDEFCK_60_, _82_GPSLPREVDVITATLPLDQQVDIVNEVMR_110_, _88_EVDVITATLPLDQQVDIVNEVMR_110_, _114_NEPFDENLVSK_124_ and _145_GSSWCSFSYEPK_156_. The peptide sequences matched with those predicted for CsTegu20.6 with a sequence coverage of 56% (Table S2).

### 2.10. Stage-Specific Expression

**Figure 9:**
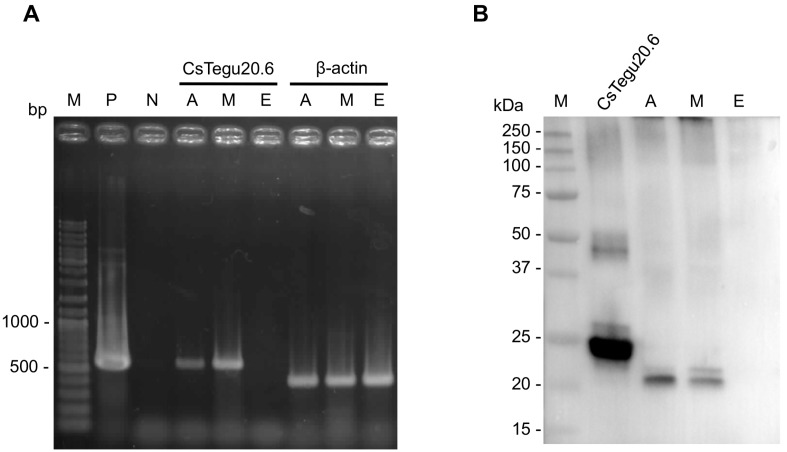
Transcriptional and protein analysis of CsTegu20.6 across *C. sinensis* developmental stages. (**A**) PCR was performed using specific primers for the complete coding sequence of CsTegu20.6; (**B**) expression of the native CsTegu20.6 was confirmed in adult worm and metacercariae using anti-rCsTegu20.6 sera. M, DNA markers; P, positive control; N, negative control; A, adult worm cDNA library; M, metacercariae cDNA library; E, egg cDNA library.

PCR experiments showed that an amplicon specific to CsTegu20.6 was detected in the adult worm and metacercariae of *C. sinensis*, but not in the egg (Figure 9A). A full-length cDNA clone of CsTegu20.6 was used as the positive control. We also confirmed that native CsTegu20.6 was expressed in adult worm and metacercariae using anti-rCsTegu20.6 sera (Figure 9B). As previously described in Figure 5C, CsTegu20.6 was predicted to form homodimers, which can be an unidentified protein complex of molecular weight between the 37-kDa and 50-kDa bands. The lack of expression in the egg possibly arises from the fact that tegumental proteins are differentially expressed in the body according to developmental stages [55]. By the manipulation of its biological aspects, the tegument serves as an important biological means of protection. When encysted metacercariae migrate to the common and hepatic bile ducts in the mammalian host and then grow to adult worms, the juvenile worms are completely exposed to the host immune attack and toxic bile acids during the processes of migration and development [56]. Conversely, similar expression between the adult worm and metacercariae suggests that CsTegu20.6 might be useful as a potential antigen for the early diagnosis of clonorchiasis, which would have the advantage that *C. sinensis* infections could be detected prior to egg deposition [57].

### 2.11. Immunolocalization in Adult C. sinensis

**Figure 10:**
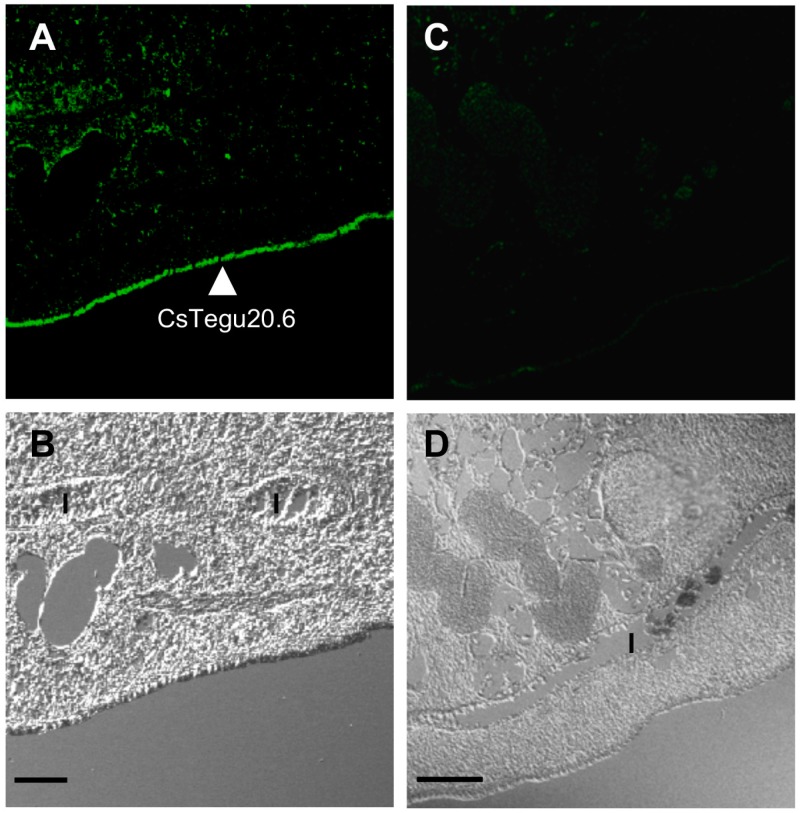
Immunohistochemical localization of CsTegu20.6 in *C. sinensis* adult worm. An adult fluke (×100) stained with 1:200 diluted FITC-conjugated anti-rat IgG antibody showed intense staining in the outer surface of the tegument ((**A**,**B**), closed arrowhead); Non-immunized rat sera were used to make a negative control (**C**,**D**). The upper images showed the tegument and underlying tissues of adult worm under fluorescence microscope. The lower images showed the same part under optical microscope. Scale bars indicate 50 μm. I, intestine.

As expected, immunolocalization showed that CsTegu20.6 was dominantly distributed at the tegument of the *C. sinensis* adult worm, using anti-CsTegu20.6 sera as the primary antibody and fluorescein isothiocyanate (FITC)-conjugated anti-rat IgG as the secondary antibody (Figure 10A,B). In contrast, no staining was observed in sections incubated with the pre-immunized serum (Figure 10C,D).

## 3. Materials and Methods

### 3.1. Sequence Identification and Characterization

CsTegu20.6 was searched against an expressed sequence tag (EST) database of *C. sinensis* developmental stages constructed using large-scale sequencing [18,58]. The nucleotide sequence was translated using the ExPASY Translate tool (http://web.expasy.org/translate/). The ORF was predicted using the ORF-predictor server [59]. The physico-chemical properties of the predicted protein, including molecular weight and pI, were calculated by ProtParam [60]. Functional domains was searched using InterProScan [61], signal peptides with SignalP 3.0 [62], protein subcellular localization using TargetP [63] and transmembrane regions with TMHMM [64]. Homologous proteins were collected from a BLASTP search (National Center for Biotechnology Information, Bethesda, MD, USA) [65]. Multiple sequence alignments were generated using MAFFT [22] and were displayed by ESPript [21].

### 3.2. Combined 3D Modeling Methods and Refinement

Although the 3D structure of CsTegu20.6 was modeled using Swiss-Model [23] and I-TASSER [24], the optimal 3D structure was not constructed because of the unavailability of appropriate templates. Therefore, we used a modified method to obtain the full-length and refined structures of the CsTegu20.6 protein (Figure 1) as follows: (1) the CsTegu20.6 protein sequence was split into D1 and D2 domains based on the disordered region predicted by DisoPred3 [66] (Figure 1A); the C1 fragment was selected as a bridge in order to later combine the two domains; (2) the 3D structures of D1, D2 and C1 were modeled using I-TASSER [24] (Figure 1B); (3) the residue numbers of the D2 domain and the C1 fragment were readjusted to the original numbers in the sequence, renaming them as D2′ and C1′, respectively. Each domain and C1′ fragment was superposed using the TM-Score [29]. Both D1 and D2′ were aligned to the C1′ fragment using the TM-Score so that D1 and D2′ were correctly oriented 5′ to 3′ with respect to each other. Then, the full-length structure was obtained by combining each superposed structure, from which the C1′ fragment was removed manually (Figure 1C); (4) The low free-energy conformations of the combined full-length structure were refined by full-atomic simulations using ModRefiner [30] (Figure 1D). The additional refinement process was performed in two stages. First, the loop regions aa13–20, aa74–88 and aa145–154 were refined by GalaxyLoop [32] using the ‘PS1tbm protocol’ scoring method. Then, both the backbone and side chain of the structure were refined using GalaxyRefine [33] incorporating the “both mild and aggressive relaxation” method.

### 3.3. Model Evaluation and Quality Assessment

Structural evaluation and stereochemical quality assessment were performed using several evaluation and validation tools both before and after refinement. The potential errors in the 3D models were evaluated using Ramachandran plots [34] obtained from PROCHECK [67], ProSA [35], QMEAN [36] and ERRAT [37].

### 3.4. Structural Comparison and Visualization

Secondary structure elements and the structure topology of CsTegu20.6 were analyzed using ProFunc [68]. The deconSTRUCT [40] and DALI [42] were employed to detect the aligned regions of proteins based on structural similarities. The oligomeric state of the structure was predicted using GalaxyGemini [44], with subsequent energy minimization. All structure visualization was performed using UCSF Chimera [69] and PyMOL v.099rc6 (http://www.pymol.org).

### 3.5. Protein-Compound Interaction and Drug-Likeness

COACH [47] was used to predict calcium-binding sites in the N-terminal region of CsTegu20.6, based on the identification of analogs with similar binding sites. GalaxySite [48] was employed to predict specifically binding non-metal ligands and their binding conformations for screening putative inhibitors of CsTegu20.6. The detailed information regarding specific protein-compound interactions such as hydrophobic interactions, was visualized using LigPlot+ [70] implemented in GalaxySite. ADMET properties were evaluated using admetSAR [49]. Lipinski’s rule of five and binding energies were calculated using the Molinspiration online server (http://www.molinspiration.com/cgi-bin/properties) and MTiAutoDock [71], respectively.

### 3.6. Ethics Statement

The animal care and use protocol was reviewed, and the experiments were approved by the Institutional Animal Care and Use Committee (IACUC) at Korea National Institute of Health (KNIH) (Approval Nos. NIH-06-15, NIH-07-16 and NIH-08-19). The experiments were approved by the Committee on the Ethics of Animal Experiments of the Korean Centers for Disease Control and Prevention (KCDC) (Korean Laboratory Animal Act No. KCDC-122-14-2A). The use of experimental animals was maintained and handled in strict accordance with institutional guidelines at KCDC. The serum samples were collected from experimental rats.

### 3.7. Adult Worms and Sera

*C. sinensis* metacercariae were obtained from naturally-infected fish, *Pseudorasbora parva*, caught in Jin-Ju, South Korea, using previously published methods [72]. We administered 500 metacercariae orally twice to each New Zealand white rabbit (Orient Bio Inc., Seongnam, Korea), and adult worms were collected from the bile ducts of the infected rabbits at 2 months post-infection. Then, the worms were prepared for immunohistochemistry.

### 3.8. Plasmid Construction

The full-length CsTegu20.6 gene sequence was isolated from an EST library of adult worms using polymerase chain reaction (PCR). C1000 Touch^TM^ Thermal Cycler (Bio-Rad Laboratories Inc., Hercules, CA, USA) and TaKaRa LA Taq polymerase (TAKARA, RR042, Shiga, Japan) were used in the PCR reaction. The forward and reverse primers for CsTegu20.6 were 5′-GGG CAA GGT ACC ATG GAG CCA TTC TTA GAA G-3′ and 5′-CCC GTT AAG CTT TCA GCT TGG TGT CTT CCA C-3', incorporating *Kpn* I and *Hind* III restriction sites (underlined), respectively. Cycling conditions were as follows: 95 °C for 30 s, followed by 30 cycles of 94 °C for 30 s, 58 °C for 30 s, 72 °C for 60 s and, finally, 72 °C for 10 min. The amplified PCR products were purified, digested with *Kpn* I and *Hind* III endonucleases, run on a 1% agarose gel, excised from the gel and ligated into the bacterial expression vector pRSETb (Invitrogen, Carlsbad, CA, USA). After antibiotic selection, positive clones were confirmed by nucleotide sequencing (Macrogen, Seoul, Korea) and transformed into *Escherichia coli* BL21 (DE3) pLysS (Invitrogen).

### 3.9. Expression and Purification

Recombinant fusion protein was expressed by isopropyl-β-d-thiogalactopyranoside induction at a final concentration of 0.5 mM at 30 °C for 5 h. Bacterial cells were harvested by centrifugation at 70,000× *g* for 10 min at 4 °C. The cells were lysed by sonication in buffer (50 mM NaH_2_PO_4_, 300 mM NaCl and 10 mM imidazole), and the supernatant was collected after centrifugation at 13,000× *g* for 30 min. The fusion protein was purified by Ni-NTA affinity chromatography following the manufacturer’s instructions (Qiagen, Gaithersburg, MD, USA). The purified recombinant protein was examined by 12% sodium dodecyl sulfate-polyacrylamide gel electrophoresis (SDS-PAGE) and stained by Coomassie brilliant blue G-250.

The rCstegu20.6 proteins were subjected to 12% SDS-PAGE and subsequently transferred to PVDF membranes (Millipore, 0.45 µm, IPVH00010, Molsheim, France). Non-specific binding was blocked by incubation in blocking buffer (5% skim milk, 20 mM Tris, 500 mM NaCl, 0.05% *v*/*v* Tween 20, pH 7.4) at room temperature. The membranes were reacted with HRP conjugated anti-polyhistidine antibody (mouse, polyclonal, Abcam, ab49781, 1:2000 dilution) for 1 h. After washing with TBST, the immunoreactive bands were visualized on the membrane using 4-choloro-1-naphthol solution (Sigma, C6788, St. Louis, MO, USA).

### 3.10. Preparation of Antiserum

To obtain anti-rCsTegu20.6 sera, Sprague–Dawley rats (Orient Bio Inc., Seongnam, Korea) were used for peptide injection. The initial injection included Freund’s complete adjuvant (Sigma-Aldrich, St. Louis, MO, USA) mixed with purified rCsTegu20.6 (100 µg), and 3 subsequent injections included Freund’s incomplete adjuvant (Sigma-Aldrich) at 1-week intervals. Blood was collected 2 weeks after the final immunization, and its serum was separated as the antisera against CsTegu20.6. The specificity of the antibody was detected by Western blot as described below.

### 3.11. Analysis of Protein Expression during C. sinensis Developmental Stages

Adult worms, metacercariae and eggs of *C. sinensis* were homogenized in lysis buffer (40 mM Tris, 7 M urea, 2 M thiourea and 4% CHAPS), respectively. The concentration of each crude extract was determined by the Bradford assay (Bio-Rad Laboratories, Berkeley, CA, USA). A 10-μg sample of each crude extract was subjected to 12% SDS-PAGE and electrotransferred to a polyvinylidene fluoride membrane. The anti-CsTegu20.6 serum (1:1000 dilution) and HRP-conjugated anti-rat IgG (1:2000 dilution) were used primary and secondary antibodies, respectively. Final visualization was performed using the ChemiDoc^TM^ imager (Bio-Rad).

### 3.12. Transcript Analysis of Developmental cDNA Libraries in C. sinensis

Complete ORF transcripts of CsTegu20.6 were amplified from specific stages of *C. sinensis*, including adult worms, metacercariae and eggs, using the previously described primers. PCR was performed using a thermal cycling profile of 95 °C for 1 min, 30 cycles at 94 °C for 30 s, 55 °C for 30 s and 72 °C for 1 min followed by a 72 °C extension for 10 min. The analysis of transcripts obtained from each cDNA library [18,58] was performed by 1% agarose gel electrophoresis and ethidium bromide staining.

### 3.13. Immunolocalization of CsTegu20.6 in C. sinensis Adult Worms

Immunohistochemistry was performed to investigate the localization of CsTegu20.6 within the adult worm. Fresh-washed adult worms of *C. sinensis* were fixed with 4% paraformaldehyde, dehydrated by a graded ethanol series and embedded in paraffin blocks. Sections of adult fluke (4 μm thick) were mounted on glass slides, deparaffinized, rehydrated and washed in phosphate-buffered saline (PBS). The slides were incubated with anti-CsTegu20.6 sera diluted 1:100 in PBS at room temperature for 2 h and washed several times in PBS. Non-immunized rat sera were employed as a negative control. The slides were then incubated for 2 h in FITC-conjugated anti-rat IgG (1:200 dilution, Sigma, St. Louis, MO, USA). All specimens were washed and examined using a light/fluorescence microscope (Axioplot, Carl Zeiss, Jena, Germany).

## 4. Conclusions

In this study, we identified and characterized CsTegu20.6 using in silico and molecular approaches. A reliable tertiary structure of CsTegu20.6 protein was modelled using combined 3D modeling methods because of the unavailability of appropriate templates. The CsTegu20.6 protein sequence was split into two domains based on the disordered region, and then, the structure of each domain was modeled using I-TASSER. A final full-length structure was obtained by combining two structures and refining the whole structure. Through the structure-based virtual screening, staurosporine was proposed as a putative inhibitor, which can be used as preliminary data for the development of novel anti-*C. sinensis* drugs. At the molecular level, CsTegu20.6 mRNAs were abundant in adult and metacercariae, but not in the egg. CsTegu20.6 localized to the exterior of the tegument in the adult fluke.

## Figures and Tables

**Table 1:** Alignment scores of three structures, superimposed using the deconSTRUCT server.

PDB ID	Aln. Score ^a^	Aln. Length ^b^	RMSD (Å) ^c^	Avg. dL ^d^	Genom. Z ^e^	Target Molecule
1YO3_A	67.31	78	1.95	1.20	−5.46	Dynein light chain 1
1RE6_A	66.35	78	1.98	0.20	−7.05	Dynein light chain 2
1JFJ_A	49.94	65	3.12	2.00	−3.16	Calcium-binding protein

^a^ Aln. Score is the alignment value calculated based on the closely related protein structures available in the protein databank; ^b^ Aln. Length is the length of the structure sequence aligned to the query sequence; ^c^ RMSD is the root mean square deviation; ^d^ Avg. dL is the average length mismatch for matched secondary structure elements alignments; ^e^ Genom. Z is the Z-score for the orientational match.

## Supplementary Materials

Supplementary materials can be found at www.mdpi.com/1422-0067/18/3/557/s1.

## Abbreviations

STU	Staurosporine
ANP	Phosphoaminophosphonic acid-adenylate ester
BK3	3-(naphthalen-1-ylmethyl)-1-(piperidin-4-ylmethyl)-1H-pyrazolo[3,4-d]pyrimidin-4-amine
CRK	4-{(z)-[2-[3-(methylsulfanyl)propanoyl]-5-oxo-1-(2-oxoethyl)-1,5-dihydro-4h-imidazol-4-ylidene]methyl}benzenolate
DTQ	4-[3-hydroxyanilino]-6,7-dimethoxyquinazoline
MRD	(4r)-2-methylpentane-2,4-diol
