# Supplementary material for: Molecular and Structural Characterization of the Tegumental 20.6-kDa Protein in Clonorchis sinensis as a Potential Druggable Target
Source: Int J Mol Sci. 2017 Mar 4;18(3):557. doi: 10.3390/ijms18030557 (PMC5372573; doi:10.3390/ijms18030557)
Supplement: Supplementary file 1 [file ijms-18-00557-s001.pdf]

Supplementary Materials: Molecular and Structural Characterization of the Tegumental 20.6-kDa Protein in *Clonorchis sinensis* as a Potential Druggable Target

Yu-Jung Kim, Won Gi Yoo, Myoung-Ro Lee, Jung-Mi Kang, Byoung-Kuk Na, Shin-Hyeong Cho, Mi-Yeoun Park and Jung-Won Ju

Table S1. Predicted molecular pharmacokinetic properties of the inhibitor candidates.

	STU		ANP		BK3		CRK		DTQ		MRD	
	Result	Probability(%)	Result	Probability(%)	Result	Probability(%)	Result	Probability(%)	Result	Probability(%)	Result	Probability(%)
**1. ADMET**												
**Absorption**												
**BBB**	-	52.4	+	87.6	+	96.6	+	69.3	+	67.1	+	93.4
**HIA**	+	97.3	-	69.5	+	99.8	+	93.7	+	93.1	+	97.4
**Caco-2**	-	52.1	-	72.0	-	62.5	-	55.9	+	61.6	+	54.5
**Metabolism**												
**CYP450 substrate**												
**2C9**	NS	76.8	NS	85.3	NS	87.4	NS	69.2	NS	76.3	NS	82.8
**2D6**	NS	76.7	NS	83.5	NS	65.2	NS	79.8	NS	84.4	NS	85.4
**3A4**	S	79.9	NS	56.0	NS	65.1	NS	50.0	NS	57.4	NS	58.2
**CYP450 inhibitor**												
**1A2**	NI	56.3	NI	88.5	I	55.7	NI	71.5	I	59.1	NI	84.0
**2C9**	NI	67.3	NI	92.2	NI	89.8	NI	71.0	NI	61.3	NI	86.8
**2D6**	NI	82.3	NI	90.1	NI	85.3	NI	90.8	NI	61.3	NI	92.2
**2C19**	NI	70.0	NI	91.3	NI	78.1	NI	59.3	I	67.9	NI	74.3
**3A4**	NI	56.3	NI	89.6	NI	89.2	NI	81.9	NI	54.8	NI	89.7
**CYP Inhibitory Promiscuity**	I	59.0	NI	97.2	I	65.1	NI	88.0	I	73.4	NI	88.9
**Toxicity**												
**AMES toxicity**	-	63.2	-	78.0	-	59.5	-	64.3	+	84.5	-	86.8
**FISH**	-	53.7	-	60.4	+	95.9	+	86.0	-	87.9	-	89.1
**Honey Bee**	-	72.4	-	68.9	-	83.8	-	74.9	-	72.2	+	78.4
**Carcinogens**	-	85.7	-	91.1	-	82.8	-	91.6	-	94.3	+	68.2
**Acute Oral Toxicity**	III	58.0	III	63.4	III	72.8	III	65.0	III	54.5	III	84.2
**2. Lipinski Molecular Descriptor**												
**HBA (≤10)**		7.0		18.0								
**HBD (≤5)**		2.0		9.0								
**clongP (≤5)**		3.9		-3.7								
**MW (≤500)**		466.5		506.2								
**Additional Molecular Descriptor**												
**n-Rot (≤10)**		2.0		8.0								
**TPSA (≤140)**		69.5		281.9								
**Binding Energy (kcal/mol)**		-7.2		-11.0								

BBB, blood-brain barrier; HIA, human intestinal absorption; + positive; - negative; I, inhibitor; NI, non-inhibitor; NS, non-substrate; HBA, number of hydrogen bond acceptors; HBD, number of hydrogen bond donors; clogP, logarithm of compound partition coefficient between n-octanol and water; MW, molecular weight; n-Rot, number of rotatable bonds; TPSA, topological polar surface area. A compound is predicted as a class III risk for acute toxicity when the LD_{50} is greater than 500 mg/kg.

<Inhibitor candidates>
STU: staurosporine
ANP: phosphoaminophosphonic acid-adenylate ester
BK3: 3-(naphthalen-1-ylmethyl)-1-(piperidin-4-ylmethyl)-1H-pyrazolo[3,4-d]pyrimidin-4-amine
CRK: 4-{(z)-[2-[3-(methylsulfanyl)propanoyl]-5-oxo-1-(2-oxoethyl)-1,5-dihydro-4h-imidazol-4-ylidene]methyl}benzenolate
DTQ: 4-[3-hydroxyanilino]-6,7-dimethoxyquinazoline
MRD: (4r)-2-methylpentane-2,4-diol

Table S2. LC-MS/MS identification of rCsTegu20.6.

Name	Mass	Score	Queries matched	Sequence coverage	Matched peptide	Start	End
CsTegu20.6	20565	489	149	56%	NNIDPSMIKRWQVLFDADDSGVITLDEFCK	31	60
					WQVLFDADDSGVITLDEFCK	41	60
					GPSLPREVDVITATLPLDQQVDIVNEVMR	82	110
					EVDVITATLPLDQQVDIVNEVMR	88	110
					NEPFDENLVSK	114	124
					GSSWCSFSYEPK	145	156

```
1   atggagccattcttagaagccttttttagcattgacacggaccacacagagaggatcact
     M  E  P  F  L  E  A  F  F  S  I  D  T  D  H  T  E  R  I  T
61  atacgggagctgcaagactatgtgaggcgaaataatattgatccgtcaatgattaagcga
     I  R  E  L  Q  D  Y  V  R  R  N  N  I  D  P  S  M  I  K  R
121 tggcaagttttattcgacgccgacgattccggagtgattacactggatgaattttgcaag
     W  Q  V  L  F  D  A  D  D  S  G  V  I  T  L  D  E  F  C  K
181 acgcttggaattcgtccctctgaagcccgggcttacaacgcaaatatggttcgagctagt
     T  L  G  I  R  P  S  E  A  R  A  Y  N  A  N  M  V  R  A  S
241 cgtggtccttcgctgccacgcgaggttgacgttattactgcaactctgcctttggaccaa
     R  G  P  S  L  P  R  E  V  D  V  I  T  A  T  L  P  L  D  Q
301 caggttgatattgtcaatgaggtgatgcgactgacgcgcaatgaacccttttgatgagaat
     Q  V  D  I  V  N  E  V  M  R  L  T  R  N  E  P  F  D  E  N
361 ctggtaagcaaacaactgaagcaatttctcgaccgtcaatacggacgaatgtggcatgtg
     L  V  S  K  Q  L  K  Q  F  L  D  R  Q  Y  G  R  M  W  H  V
421 gtgataacaaaaggatccagctggtgcagtttctcttacgagccgaagacctcacttttc
     V  I  T  K  G  S  S  W  C  S  F  S  Y  E  P  K  T  S  L  F
481 ttccaactgcgcaaatacacctaccttgtgtggaagacaccaagc
     F  Q  L  R  K  Y  T  Y  L  V  W  K  T  P  S
```

Figure S1. Characterization of CsTegu20.6 sequence. CsTegu20.6 cDNA (528 nt) and deduced amino acid sequence (175 aa) are displayed. The shadowed fragment indicates the EF-hand calcium-binding domain (PS00018) and the underlined fragment represents the dynein light-chain domain (PF01221).

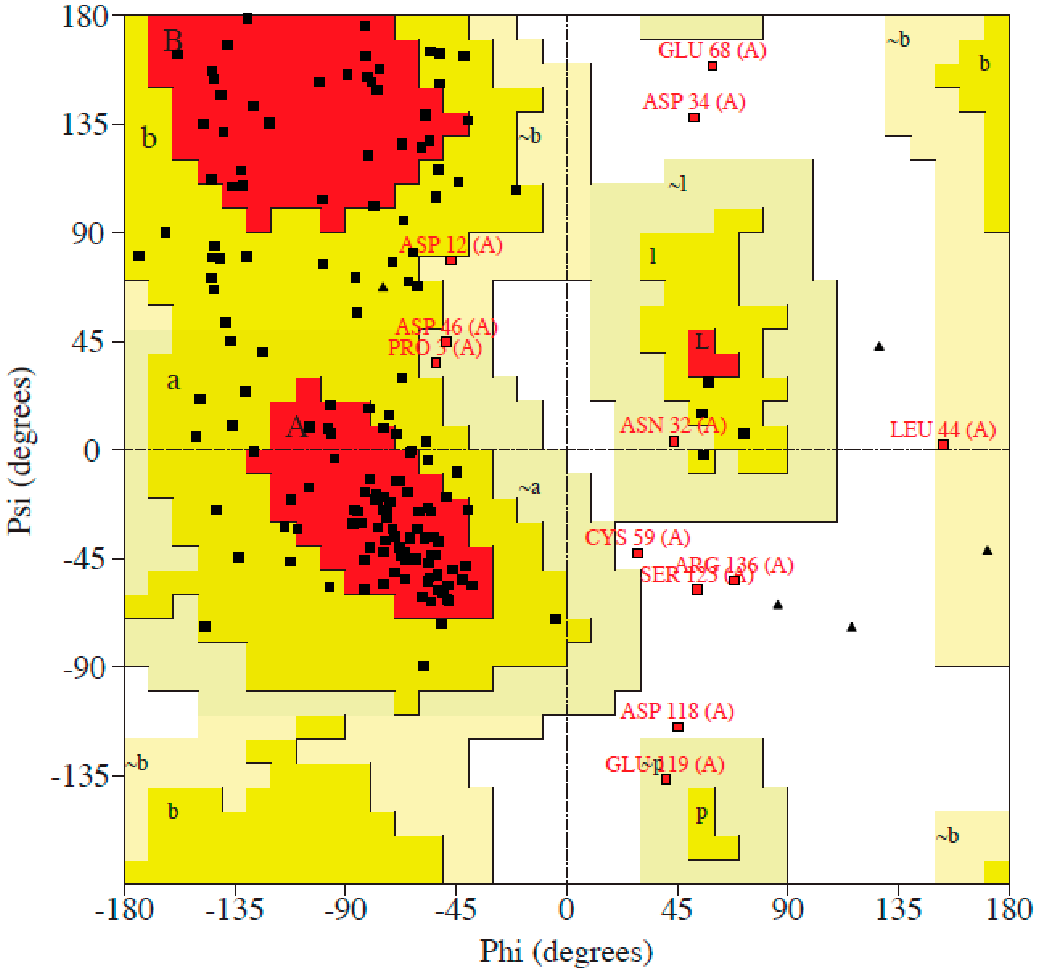

Plot statistics

Residues in most favoured regions [A,B,L]	103	64.8%
Residues in additional allowed regions [a,b,l,p]	45	28.3%
Residues in generously allowed regions [~a,~b,~l,~p]	5	3.1%
Residues in disallowed regions	6	3.8%
	----	------
Number of non-glycine and non-proline residues	159	100.0%
Number of end-residues (excl. Gly and Pro)	2	
Number of glycine residues (shown as triangles)	5	
Number of proline residues	9	

Total number of residues	175	

Figure S2. Evaluation of 3D model of CsTegu20.6 after using I-TASSER and FG-MD. Ramachandran plot showed the residues in the most favored regions (64.8%), additional allowed regions (28.3%), generously allowed regions (3.1%), and disallowed regions (3.8%). Red (A, B, L), yellow (a, b, l, p) and light yellow (~a, ~b, ~l, ~p) indicate the most favored regions, allowed regions and generously allowed regions. White shows disallowed regions. All non-glycine and non-proline residues are shown as closed black squares while glycines (non-end) are shown as closed black triangles. Disallowed residues are colored in red.

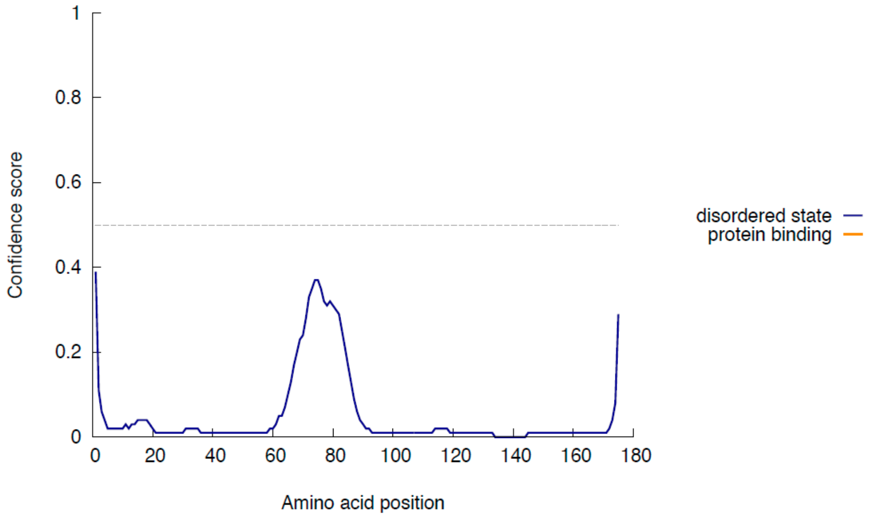

Figure S3. Disorder profile plot of CsTegu20.6. The plot indicates the position of each amino acid sequence against the disordered state. The disordered region is located between positions 61–90.

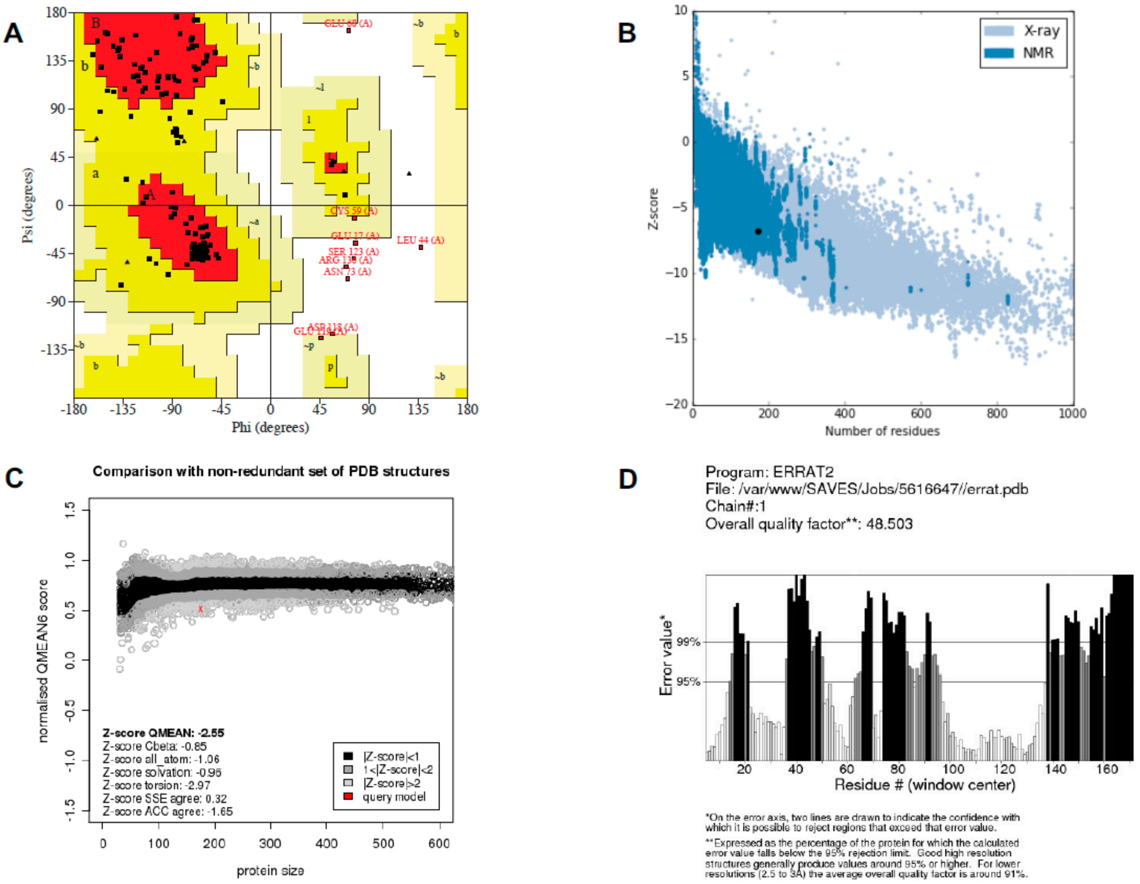

Figure S4. Validation of the initial 3D model of CsTegu20.6. (**A**) Ramachandran plot showing the residues in the most favored regions (81.8%), additional allowed regions (15.1%), generously allowed regions (0.6%), and disallowed regions (2.5%). Red (A, B, L), yellow (a, b, l, p) and light yellow (~a, ~b, ~l, ~p) indicate the most favored regions, allowed regions and generously allowed regions. White shows disallowed regions. All non-glycine and non-proline residues are shown as closed black squares while glycines (non-end) are shown as closed black triangles. Disallowed residues are colored in red. (**B**) The ProSA energy profile indicates that the Z-score was −6.75. (**C**) The QMEAN Z-score is −2.55. (**D**) In the ERRAT plot, the overall quality factor is 48.50%.

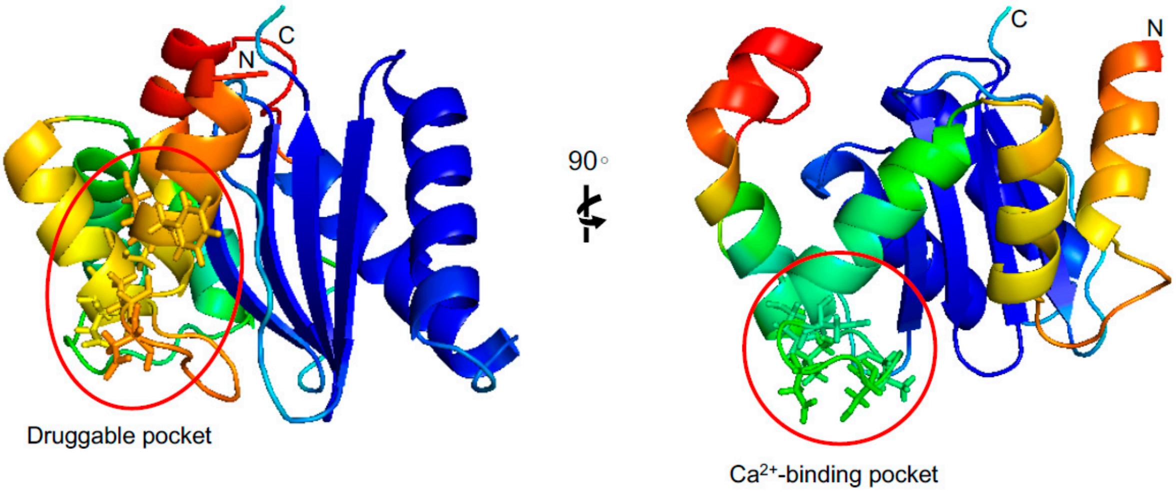

Figure S5. 3D view of residue error in CsTegu20.6 3D model. 3D cartoon of the model was colored by the per-residue error according to the B-factor values using ModFOLD6. Decreasing magnitudes, as B-factor values, are color coded from red to blue. The two faces of CsTegu20.6 are presented by vertical rotation of 90°. Druggable pocket (See the '*2.8. Virtual Inhibitor Screening*' section) and $Ca2^{+}$-binding pocket (See the '*2.7. EF-hand Calcium-binding Site*' section).
